# Supplementary material for: Dual targeting of CDK6 and LSD1 is synergistic and overcomes differentiation blockade in AML
Source: EMBO Mol Med. 2025 Aug 29;17(10):2632–60. doi: 10.1038/s44321-025-00296-2 (PMC12514269; doi:10.1038/s44321-025-00296-2)
Supplement: Supplementary file 1 — Table EV1 [file 44321_2025_296_MOESM1_ESM.pdf]

Table EV1. List of adult AML patient samples and their characteristics

Patient No.	FAB	Sex	Age (years)	Origin	Status at sample collection	Blasts (%)	Karyotype	Translocation	Mutation status
1*	M1	F	66	PB	Relapse	83	Normal		FLT3-ITD, NPM1, DNMT3A, SMC3, WT1
2*	M5	M	54	PB	Diagnosis	83	46,XY,t(9;11)(p22;q23)[25]	KMT2A-MLLT3	ATM, DAXX, JAK3, KIF17, MAML1, NOTCH3
3*	M2	M	70	PB	Relapse	84	Normal		FLT3-ITD, NPM1, FLT3-D835H, TET2, WT1
4*	M4	F	74	PB	Relapse	91	Normal		FLT3-ITD, NPM1, DNMT3A, TET2
5*	M1	M	68	PB	Relapse	73	45,XY,-7,del(20)(q11)[9] / 46,XY,der(22)t(1;22)(q11;p11)[4] / 46,XY,der(17)t(1;17)(q11;p11)[1] / 46,XY[3]		
6	M1	F	44	BM	Diagnosis	44	45,XX,der(3),-7[20]		TET2, ASXL1, RAS, RUNX1, IKZF1, BARD1
7	M2	M	69	BM	Diagnosis	74	Normal		FLT3-ITD, NPM1, TET2
8	M1	F	65	BM	Diagnosis	84	Normal		FLT3-ITD, NPM1, DNMT3A, SMC3
9	M4	F	73	BM	Diagnosis	84	Normal		FLT3-ITD, NPM1, DNMT3A, TET2
10	M1	M	64	BM	Diagnosis	96	Normal		FLT3-ITD, NPM1, TET2, DNMT3A
11	M1	M	72	BM	Relapse	98	47,XY,inv(5)(q15q32),+8[8] / 47,idem,+add(5)(p14),-inv(5)(q15q32),add(13)(q34)[8] / 46,XY[4]		FLT3-ITD
12	M5	F	48	BM	Relapse	87	Normal		FLT3-ITD, NPM1, FLT3-TKD, DNMT3A, IDH2, ZRSR2, MYBL2
13	M1	M	71	BM	Diagnosis	93	Normal		FLT3-ITD
14	M1	M	27	BM	Diagnosis	94	46, XY, t(17;19)(q23;q13), del(22)(q11q13) [20]. ish t(17;19)(WCP17+,WCP19+;WCP19+,WCP17+), del(22)(WCP22+)[2]	t(17;19)	FLT3-ITD
15	M5	M	50	BM	Diagnosis	88	Normal		FLT3-ITD, NPM1, DNMT3A, NOTCH4, PTPN11, NOTCH2, BCORL1
16	M1	M	50	BM	Diagnosis	86	Normal		FLT3-ITD
17	M4	F	74	BM	Diagnosis	79	Normal		FLT3-ITD, NPM1, DNMT3A, APC, BARD1, NFE2
18	M4	M	67	BM	Diagnosis	83	Normal		FLT3-ITD, NPM1
19	M5	M	63	BM	Diagnosis	76	Normal		FLT3-ITD, NPM1
20	M1	F	69	BM	Diagnosis	87	Normal		NPM1, ASXL1, FLT3-TKD, TET2, KMT2, MLL3, NOTCH4
21	M5	F	28	BM	Diagnosis	92	Normal		
22	M5	M	65	BM	Diagnosis	94	45,X,-Y		NPM1
23	M4	F	28	BM	Diagnosis	82	46, XX, inv(16)(p13q22) [20]	CBFB-MYH11	
24	M1	M	59	BM	Diagnosis	96	Normal		NPM1
25	M4	M	21	BM	Diagnosis	80	47,XY,+4[19]/ 46,XY[5]		NPM1

The (*) symbol indicates the samples used for xenotransplantations (PDX AML models). M: Male. F: Female. BM: Bone Marrow. PB: Peripheral Blood.
