# Supplementary material for: Dual targeting of CDK6 and LSD1 is synergistic and overcomes differentiation blockade in AML
Source: EMBO Mol Med. 2025 Aug 29;17(10):2632–60. doi: 10.1038/s44321-025-00296-2 (PMC12514269; doi:10.1038/s44321-025-00296-2)
Supplement: Supplementary file 2 — Table EV2 [file 44321_2025_296_MOESM2_ESM.pdf]

Table EV2. List of pediatric AML patient samples and their characteristics

Patient No.	FAB	Sex	Age (years)	Origin	Status at sample collection	Circulating blasts (%)	Karyotype	Translocation	Mutation status
26	M5	F	10	BM	Diagnosis	89	49,XX,+6,+8,der(9)t(9;11)(p21;q23)del(9)(q13q31)inv(9)(p24q31),der(11)t(9;11)(p21;q23),+12[10]	KMT2A-MLLT3	
27	M4	F	3	BM	Diagnosis	89	46,XX,t(11;19)(q23;p13.1)[22]/46,XX[2]	KMT2A-ELL	
28	M1	M	6	BM	Diagnosis	85	Normal	KMT2A-MLLT3	FLT3-ITD
29	M2	M	5	BM	Diagnosis	64	46,XY,t(8;21)(q22;q22),add(13)(q31),der(16)t(1;16)(q41;p13)[10]/46,XY,t(8;21)(q22;q22),add(16)(q11)[3]/46,XY[1]	AML1-ETO	
30	M7	F	1	BM	Diagnosis	64	46,XX,add(1)(p34),-5,del(11)(p12p15),der(12)t(?1;12)(p?34;q13),+r[9]/46,XX[11]		
31	M2	M	8	BM	Diagnosis	72	46,XY,t(8;21)(q22;q22)<14>/46,XY<1>	AML1-ETO	KIT, SMC1A
32	M2	M	9	BM	Diagnosis	70	46,XY,t(11;17)(q23;q23)<2>/46,sl,del(9)(q12q21)<16>/46,sl,del(9)(q21q34)<4>	KMT2A-CLTC	EZH2
33	M4	F	5	BM	Diagnosis	67	47,XX,+8<20>		FLT3-ITD

M: Male. F: Female. BM: Bone Marrow.
