# Supplementary material for: Dual targeting of CDK6 and LSD1 is synergistic and overcomes differentiation blockade in AML
Source: EMBO Mol Med. 2025 Aug 29;17(10):2632–60. doi: 10.1038/s44321-025-00296-2 (PMC12514269; doi:10.1038/s44321-025-00296-2)
Supplement: Supplementary file 3 — Table EV3 [file 44321_2025_296_MOESM3_ESM.pdf]

Table EV3. Integration analysis of ATAC-seq and RNA-seq after drug treatment on MV411: list of genes significantly increased both in gene expression and chromatin accessibility

Gene ID	log2FoldChange.x = mRNA expression	log2FoldChange.y = chromatin access
S100A9	8.90405626246701	2.63019083732592
LILRB1	4.01431964772789	2.46469557671761
LYZ	6.12587891068144	2.27405859962539
HS6ST1	1.59757320154332	2.16483162101639
GALNT6	2.13286761560367	2.13150801985573
NTNG2	1.4008998498242	2.12856519133959
TG	2.8215886885113	2.09720505397285
IL18BP	1.1572430031382	2.07718939868822
SGMS2	2.57419673837461	1.98884320618802
CKAP4	2.26485795815275	1.98614883527282
NLRC5	1.14759891155901	1.96898263718955
CLEC4A	2.78942934226052	1.96844260940558
VNN2	2.15125723882332	1.9634495758724
MSRA	1.1069083347966	1.92595637923074
LINC01127	4.03427260350797	1.88431529348158
FAM20A	3.42691664267879	1.87219858399627
PSAP	3.23345453486233	1.8423862174664
SRGN	1.87874357093825	1.8177310424298
SOD2	2.70798867140766	1.81249510187796
SIPA1L1	1.31380165858875	1.78524306976473
FPR2	5.27709195278635	1.74386368558487
SNX21	1.42266764214147	1.70927553121253
LRRK2	8.63776083908226	1.70337093185392
SPI1	1.23925439161284	1.69835637167815
GSAP	2.98619627936016	1.66291954665101
SLC24A4	6.2155913253474	1.65202090411452
S100A8	9.93664181470568	1.65064933804169
IPCEF1	6.78735198233576	1.63852468911221
CYB561A3	2.18557435260907	1.63119041765797
FGD4	4.84790503676008	1.62271092582095
RXRA	1.12727465696707	1.62182056348027
LINC00599	1.70499831930992	1.61121205973084
CDA	5.38782797252644	1.60543791659093
ENPP2	2.23960354486535	1.58973387973772
PTGER2	1.15775486361076	1.58568275419891
FNDC3B	1.22970812897126	1.57912577949036
COLEC12	2.71635850946853	1.57730328921842
SCG2	3.70345158744112	1.57540524965333
FCN1	12.0264517175343	1.57471225114595
CD82	1.50718244424391	1.56272521963897
AKR1C1	5.88901045642562	1.54202394569604
ADAMTS10	1.75943124603451	1.53562018596407
GAS7	3.33541473442682	1.5340140896653
LILRA5	4.94880823169791	1.52560092145042
MEIS3	3.09167123567751	1.52436836568483

SARM1	1.93018102239543	1.51602476809262
PPP1R16B	1.48502741641696	1.51381774552619
SNX29	1.26000274251297	1.49978338552988
LGR4	1.71944454646963	1.49650819014569
LPAR1	1.24476400089922	1.49464356204844
SLC2A6	3.81384261696291	1.49286161227976
ITGB2	2.37298557122438	1.489447801015
NRP1	2.36910419761641	1.48933300127539
CCL2	5.63949833864254	1.48765000701646
RBM47	3.64654519455687	1.47800034608728
DLEC1	1.56656975074473	1.47650622291084
CD14	8.22649304016432	1.46867759859609
VNN1	2.9667440784706	1.46691375357498
DTX1	6.03744711167219	1.45450621141196
THBS1	5.48256237080313	1.44027916463752
SLC8B1	1.09221193353309	1.43134230032707
PIK3R6	1.41311072843271	1.42734136391021
COL23A1	1.56328431066887	1.4170133301878
CLU	3.77655068175186	1.39261589650629
PDE6H	5.39952816707774	1.38428516385637
NAMPT	1.77931606007272	1.38115497879801
NEO1	2.01450299850503	1.37780568954218
MPZL3	2.14514980783791	1.37401519636357
LHFPL2	1.66737796797623	1.34090365610217
MYO1F	1.06676945610693	1.33538831966589
LINC00211	3.07472099985179	1.32916576352963
LRG1	2.44888642906237	1.3276042361587
DMXL2	2.26426392092278	1.32207406383495
FMN1	2.44087477344771	1.31730931268326
TNFAIP3	1.08631890144823	1.31299842476813
RAB37	1.42599156273993	1.3081622598235
ZBTB46	1.05516435933057	1.30745793378932
SEMA6B	3.7957663255754	1.29894046386693
JUP	1.75789842521852	1.29769892596414
SLA	2.42577571875021	1.25825170504066
ADGRE3	6.63071413102051	1.2268705243011
CACNA1D	3.29725806020181	1.19634174904319
RASAL1	8.90805294345205	1.19421661476639
MAFB	4.69103052595192	1.19252188550099
FGGY	1.07219470914597	1.17040319998331
PNOC	3.8573529906021	1.14398286692994
TREM1	2.64518703729783	1.14263493193751
COL16A1	3.13833428760341	1.13345032548646
LYN	1.83215430286393	1.07446574874868
PPP4R1L	1.27203691297731	1.02734440129389
HK2	1.35905250685744	1.02671045879894
SOCS3	1.68700133911127	1.01329201234598
ADGRG3	2.86583344468357	1.01024439083694
SFXN5	1.38437829334489	1.00793519515972
