# Supplementary material for: Dual targeting of CDK6 and LSD1 is synergistic and overcomes differentiation blockade in AML
Source: EMBO Mol Med. 2025 Aug 29;17(10):2632–60. doi: 10.1038/s44321-025-00296-2 (PMC12514269; doi:10.1038/s44321-025-00296-2)
Supplement: Supplementary file 4 — Table EV4 [file 44321_2025_296_MOESM4_ESM.pdf]

Table EV4. Sequences of siRNA oligonucleotides

Target gene	Species	siRNA sequence
**CDK6**	Human	5'-GGCAAAGACCUACUUCUGAAGUGUU-3'
		5'-GACCACUUACUUGGAUAAAGUUCCA-3'
		5'-ACCGAGUAGUGCAUCGCGAUCUAAA-3'
**LSD1**	Human	5'-GGUCUUAUCAACUUCGGCAUCUAUA-3'
		5'-CAAAGGAUGGGAUUUGGCAACCUUA-3'
		5'-CAUUUGAGGCUACUCUCCAACAAUU-3'
**Control**		5'-CUUCGUACGCGGAAUACUUCGAUGC-3'
