# Supplementary material for: Dual targeting of CDK6 and LSD1 is synergistic and overcomes differentiation blockade in AML
Source: EMBO Mol Med. 2025 Aug 29;17(10):2632–60. doi: 10.1038/s44321-025-00296-2 (PMC12514269; doi:10.1038/s44321-025-00296-2)
Supplement: Supplementary file 5 — Appendix [file 44321_2025_296_MOESM5_ESM.pdf]

Appendix for

Dual targeting of CDK6 and LSD1 is synergistic and overcomes differentiation blockade in AML

Lise Brault[1,2], Edwige Voisset[1,2], Mathieu Desaunay[1,2], Antonia Boudet[1,2], Paraskevi Kousteridou[1,3], Sébastien Letard[1,2], Nadine Carbuccia[1,2], Armelle Goubard[1,4], Rémy Castellano[1,4], Yves Collette[1,4], Julien Vernerey[1], Isabelle Vigon[5], Jean-Max Pasquet[5], Patrice Dubreuil[1,2], Sophie Lopez[1,2], Paulo De Sepulveda[1,2,6,*]

[1]Aix Marseille University, INSERM, CNRS, Institut Paoli-Calmettes, CRCM, Marseille, France;
[2] Signaling, Hematopoiesis and Mechanism of Oncogenesis Laboratory;
[3]CRCM's Integrative Bioinformatics platform;
[4]TrGET preclinical facility, CRCM;
[5]INSERM U1312, BRIC, Université de Bordeaux, Bordeaux, France;
[6]Member of Institut Carnot OPALE (Organization for Partnerships in Leukemia).

Table of Contents:

**Appendix Figure S1.**	Effects of the combination therapy on SKM1 and Mo7e AML models, and impact of in vitro treatments on mouse survival	*page 2*
**Appendix Figure S2.**	The combined therapy has no adverse effect on normal CD34+ hematopoietic cells	*page 3*
**Appendix Figure S3.**	Combined FLT3 and LSD1 inhibition has no impact on cell differentiation or leukemic CFC progenitors	*page 4*
**Appendix Figure S4.**	The impaired cell proliferation is sustained after removal of the combined therapy	*page 5*
**Appendix Figure S5.**	Up- and down-regulated genes in MV4-11 cells following treatments	*page 6*
**Appendix Figure S6.**	The combination restores the expression of LSD1 targets genes	*page 7*
**Appendix Figure S7.**	SPI1 signature analysis in MV4-11 treated cells	*page 8*
**Appendix Figure S8.**	Transcriptomic analysis of primary AML samples	*page 9*
**Appendix Figure S9.**	Zoom-in on ATAC-seq analysis of MV4-11 TCP-treated cells	*page 10*
**Appendix Figure S10.**	Upregulated cell death signatures and increased cell death in primary patient samples	*page 11*

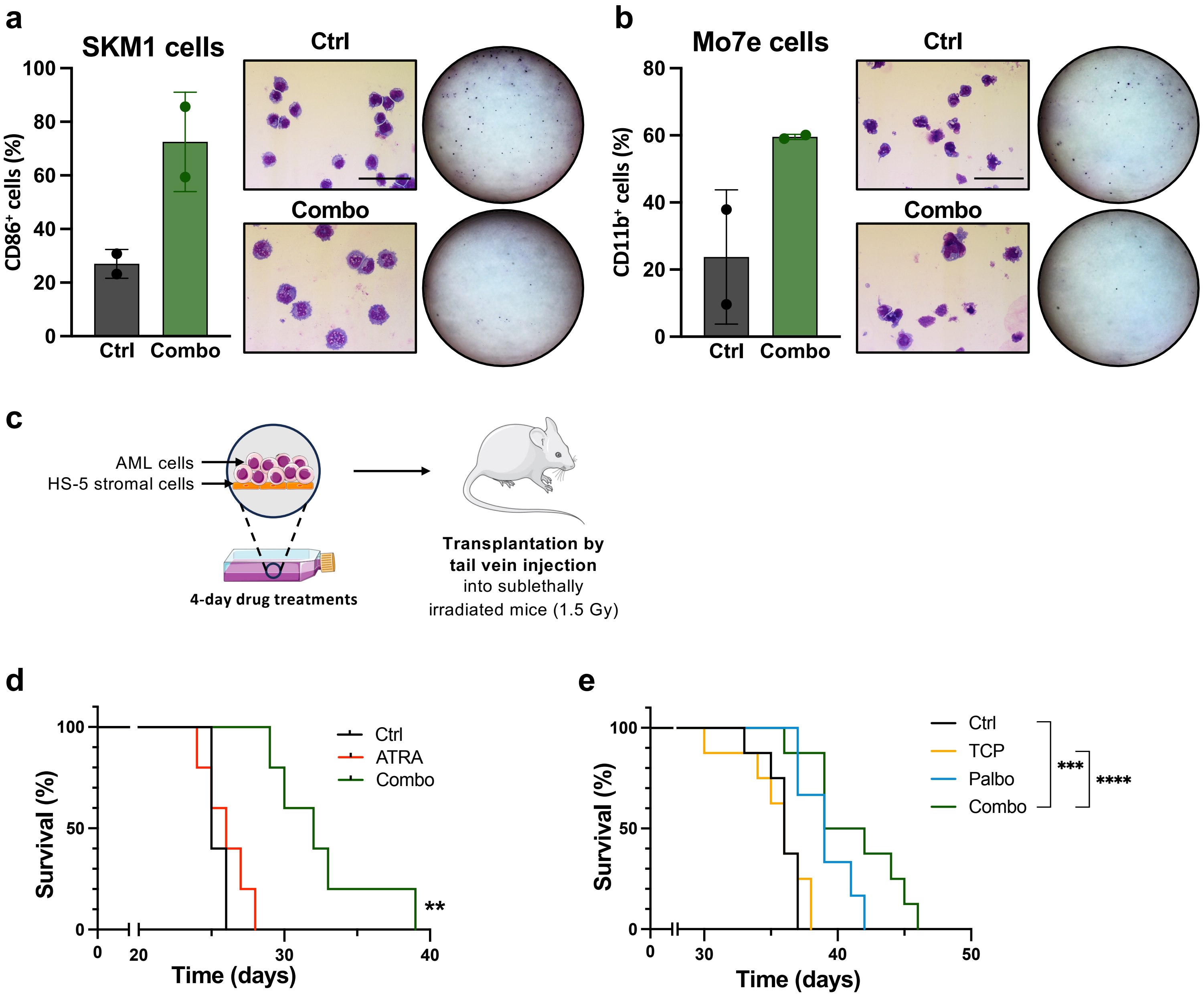

Appendix Figure S1. Effects of the combination therapy on SKM1 and Mo7e AML models, and impact of in vitro treatments on mouse survival.

(a) SKM1 cells co-cultured with HS-5 stromal cells, were either treated with vehicle control (Ctrl) or palbociclib and TCP (Combo) for 96 hours. After the treatment, cells were stained to quantify the CD86 differentiation marker (left panel), stained with May-Grünwald-Giemsa (middle panel), or seeded at an equal number on methylcellulose without additional treatment to visualize colony-forming cells (right panel).

(b) The same experiment was performed on Mo7e cells, except the CD11b differentiation marker was used instead of CD86.

(c) Experimental design of the transplantation experiment using MV4-11 cells treated in vitro.

(d) and **(e)** Kaplan-Meier survival plot of mice transplanted with MV4-11 cells treated with the combination of palbociclib and TCP (Combo), single molecules (TCP 5 µM or palbociclib 0.5 µM), or with vehicle control (Ctrl).

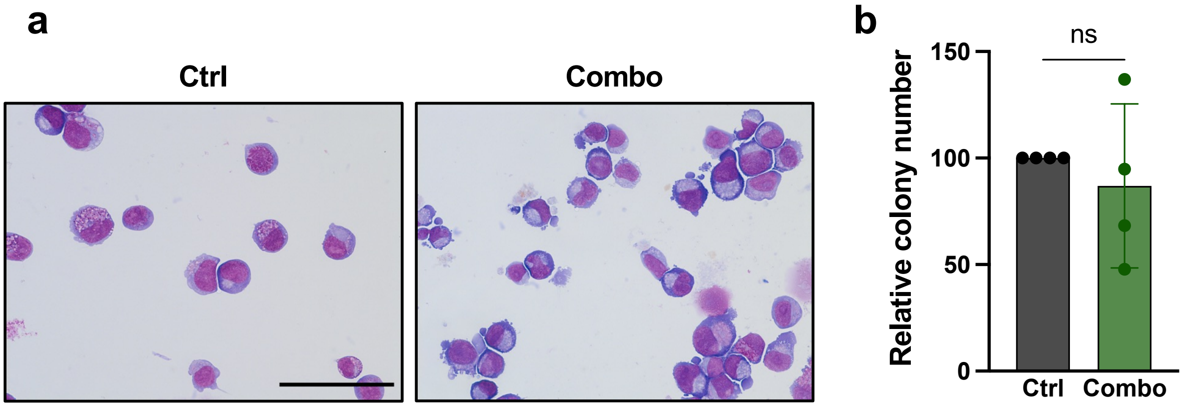

Appendix Figure S2. The combined therapy has no adverse effect on normal CD34+ hematopoietic cells.

Purified primary CD34 positive cells (n=3) were treated with palbociclib 0.5 µM and TCP 5 µM for 96 hours. After treatment, cells were stained with May-Grünwald-Giemsa **(a)**, or seeded in equal numbers (10^5 cells) in methylcellulose to quantify the number of progenitors **(b)**. Statistical analysis was performed using a paired two-tailed Student's t-test. ns is not significant.

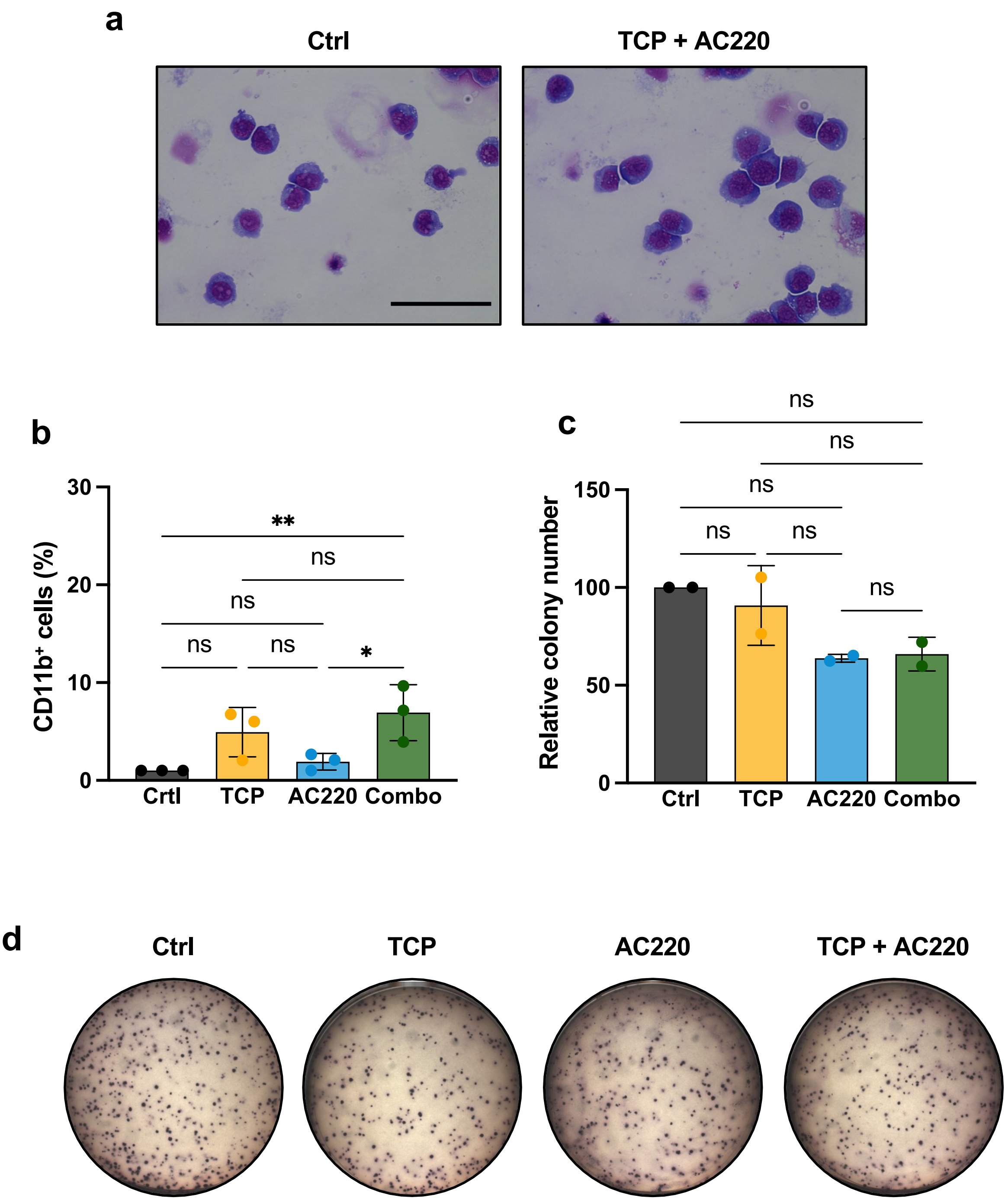

Appendix Figure S3. Combined FLT3 and LSD1 inhibition has no impact on cell differentiation or leukemic CFC progenitors.

MV4-11 cells co-cultured with HS-5 stromal cells were treated with quizartinib (AC220, 1 nM), or TCP (5 µM), or the combination of the two molecules (Combo) for 96 hours. After the treatment, cells were stained with May-Grünwald-Giemsa **(a)**, or analyzed by flow cytometry to quantify CD11b expression **(b)**, or grown in methylcellulose for 10 days **(c)**. Representative images of methylcellulose colony formation assays **(d)**. Please note that the control (Ctrl) shown here and in Expanded View 2a is the same, as both panels originate from the same experimental set. Statistical analyses were performed using a one-way ANOVA followed by Tukey's test. ns is not significant.

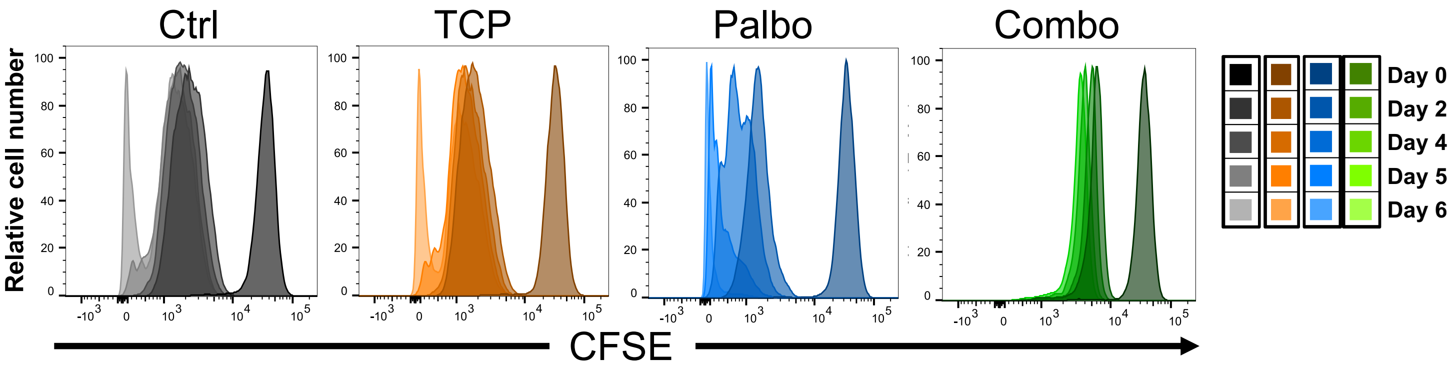

Appendix Figure S4. The impaired cell proliferation is sustained after removal of the combined therapy.
CFSE cell proliferation analysis in MOLM-14 AML cell line. Cells were treated as previously described for 96 hours, and were labeled with CFSE to monitor cell division over the following 6 days.

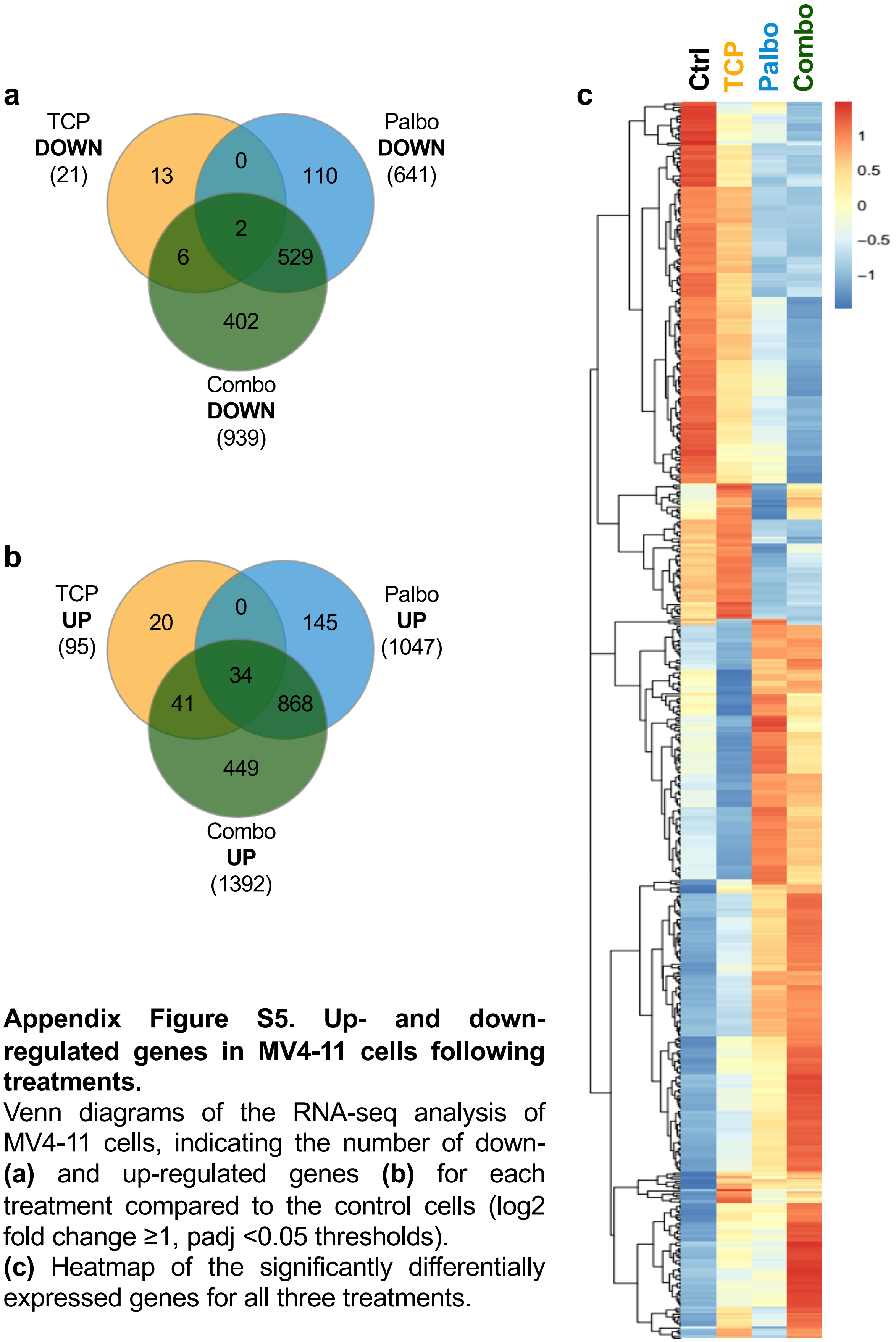

Appendix Figure S5. Up- and down-regulated genes in MV4-11 cells following treatments.
Venn diagrams of the RNA-seq analysis of MV4-11 cells, indicating the number of down- **(a)** and up-regulated genes **(b)** for each treatment compared to the control cells (log2 fold change ≥1, padj <0.05 thresholds).
(c) Heatmap of the significantly differentially expressed genes for all three treatments.

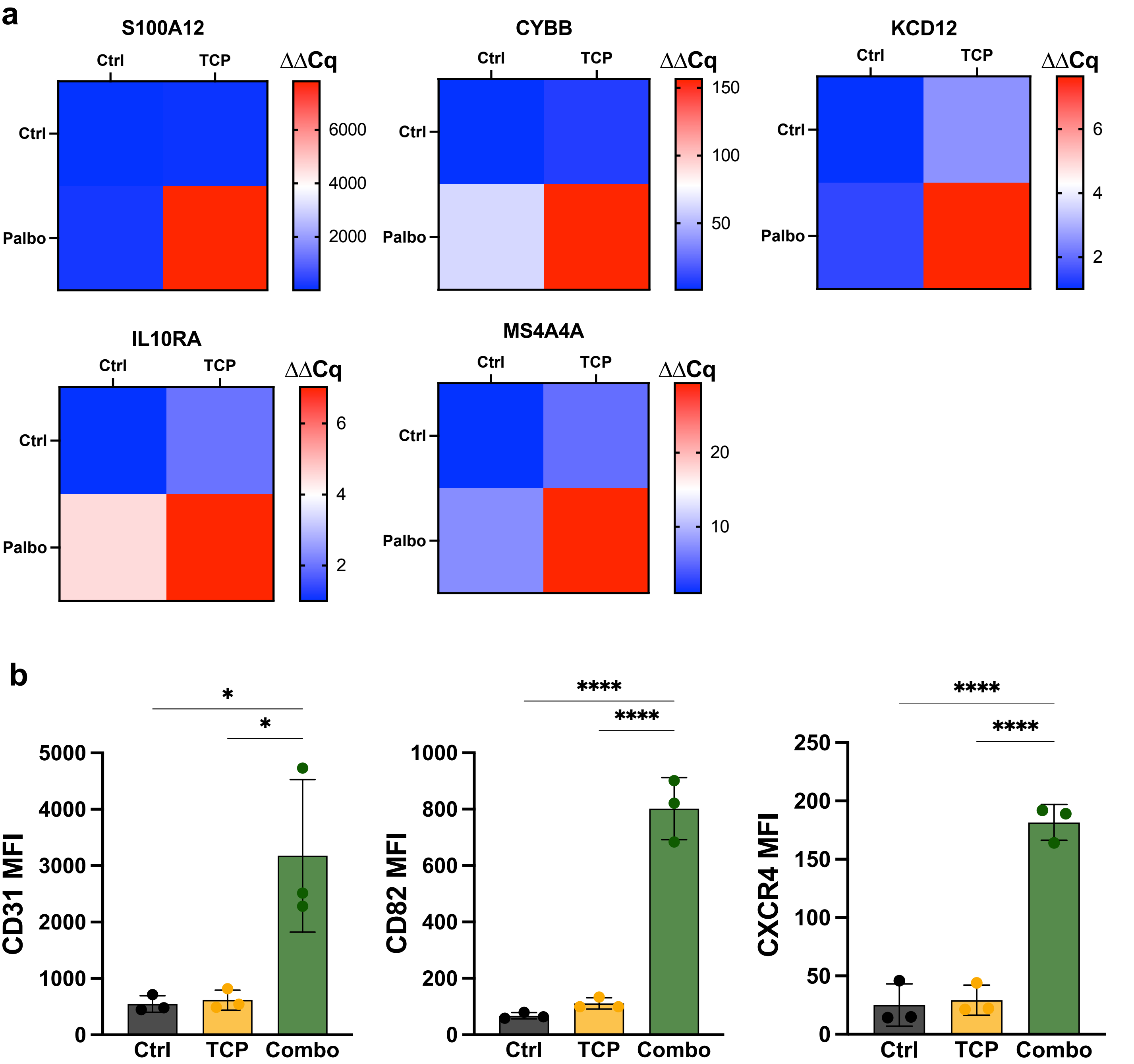

Appendix Figure S6. The combination restores the expression of LSD1 targets genes.
(a) RT-qPCR analysis of MV4-11 cells treated with the Combination (TCP+Palbo), LSD1 inhibitor (TCP), CDK6 inhibitor (Palbo) or the Control (Ctrl) for 96 hours, as indicated. RNA was extracted following the treatments to perform RT-qPCR on LSD1 downstream targets, S100A12, CYBB, KCD12, IL10RA and MS4A4A. Data are normalized and represented as DeltaDeltaCq.
(b) Quantification of CD31, CD82 and CXCR4 protein expression on MV4-11 cells treated with the Combination (Combo), LSD1 inhibitor (TCP), or control media (Ctrl). After treatment, cells were analyzed by flow cytometry. Statistical analysis was done using One-Way ANOVA.

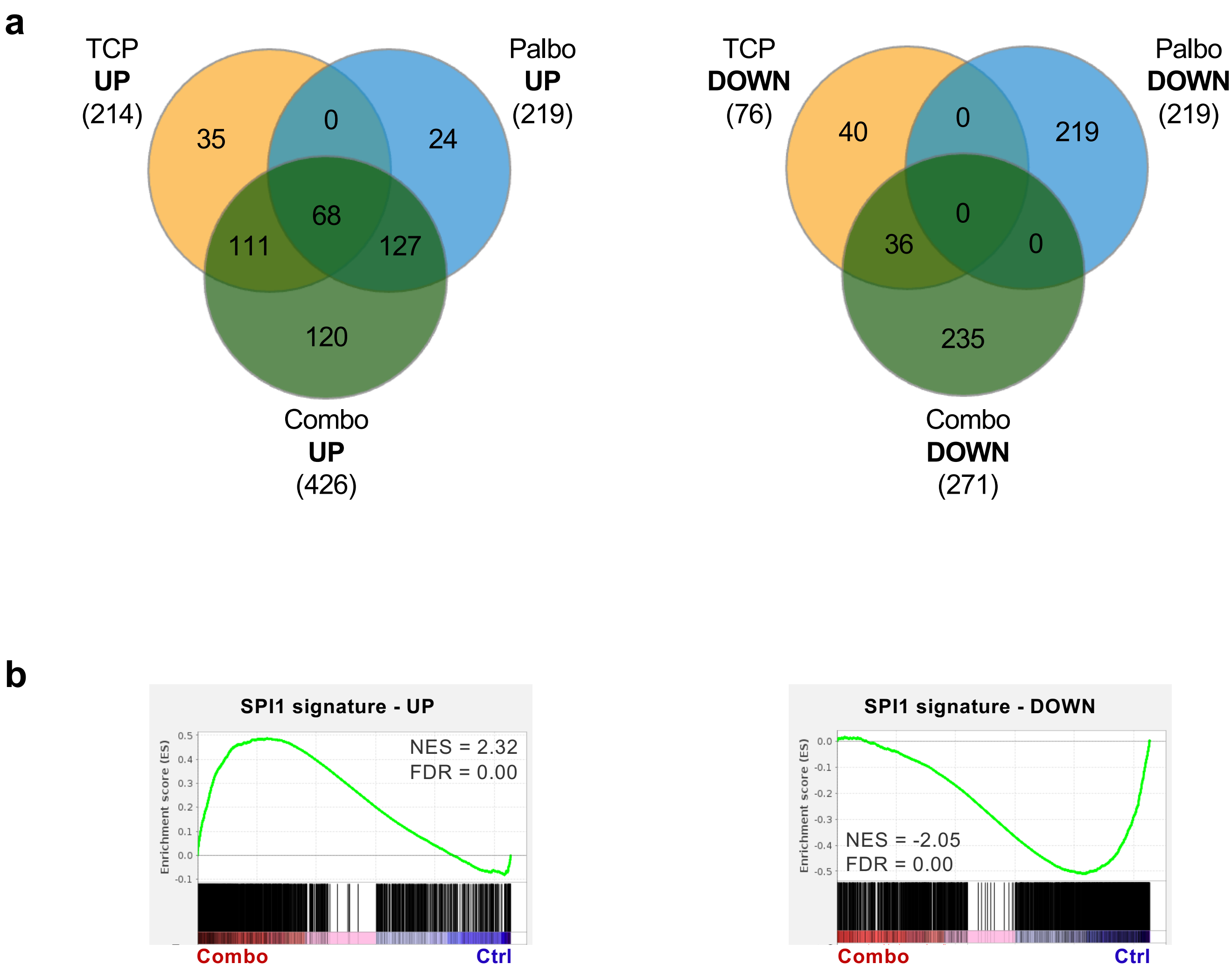

Appendix Figure S7. SPI1 signature analysis in MV4-11 treated cells.
Two SPI1/PU.1 signatures corresponding to genes upregulated by SPI1 (SPI1 UP; 1,349 genes) and genes downregulated (SPI1 DOWN; 1,779 genes) were analyzed on the MV4-11 RNA-seq datasets.
(a) Venn diagrams indicating the number of genes in SPI1 UP and SPI1 DOWN signatures significantly modified by the three treatments.
(b) GSEA comparing the gene enrichment of the combination treated cells to control cells for each SPI1 signature.

a

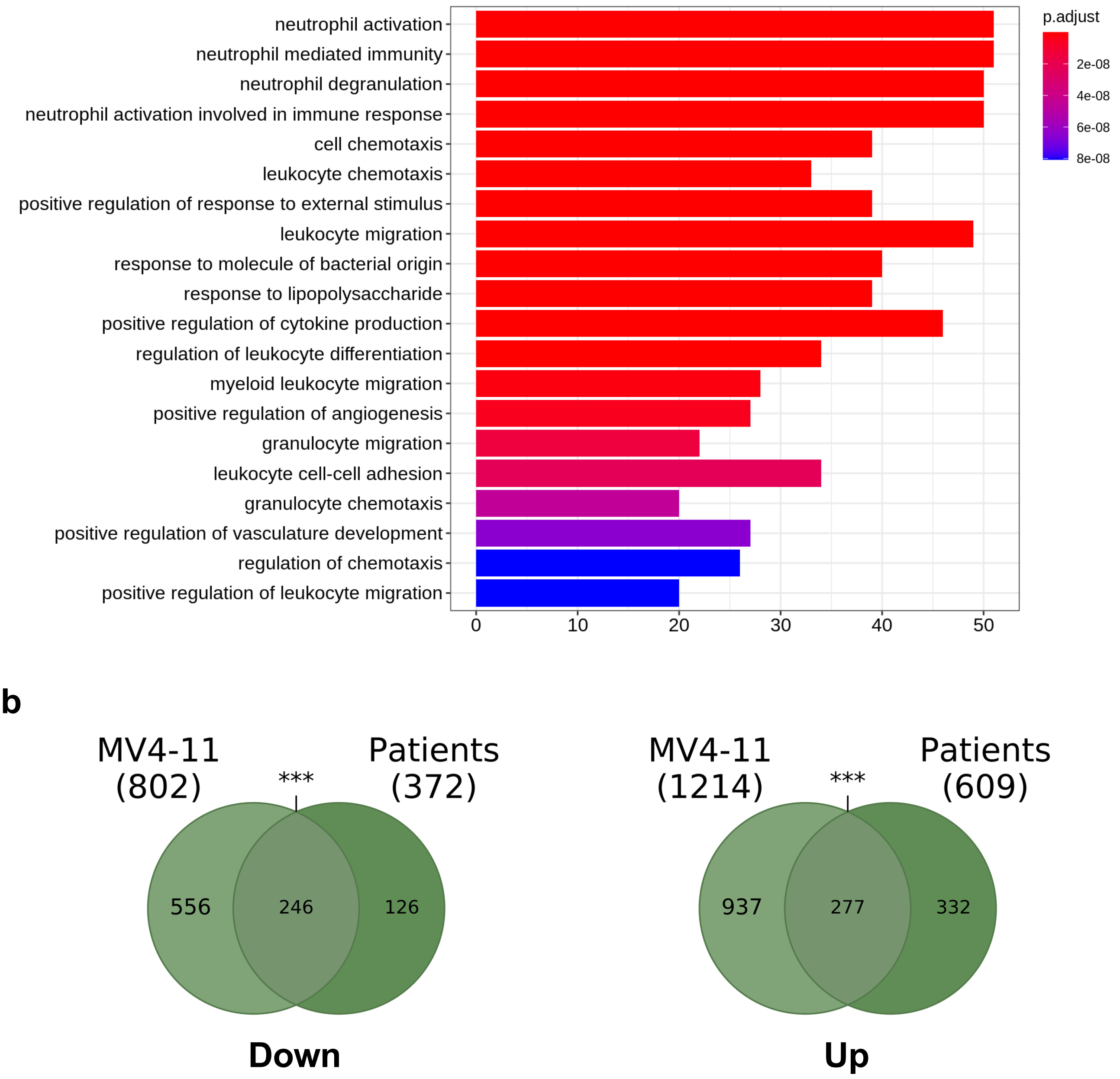

Appendix Figure S8. Transcriptomic analysis of primary AML samples.
(a) GO biological processes increased by the combined treatment of primary AML samples.
(b) Commonly deregulated genes in both MV4-11 and primary AML samples.

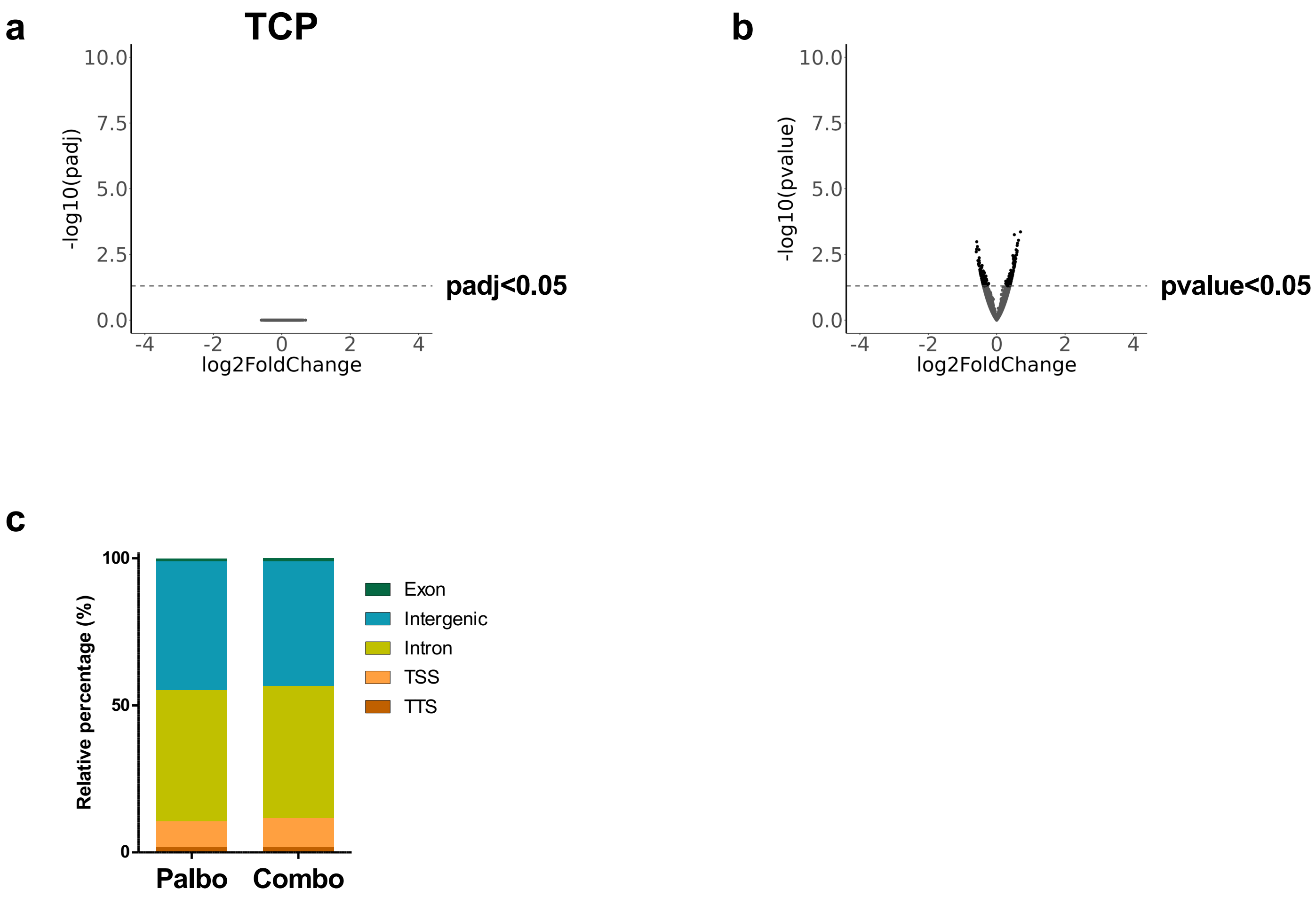

Appendix Figure S9. Zoom-in on ATAC-seq analysis of MV4-11 TCP-treated cells.
(a-b) Volcano plots of ATAC-seq analysis of MV4-11 cells treated with TCP (5 µM). **(a)** The Volcano plot is the same as the one shown in Expanded View Figure 5a and illustrates that no DNA sequence was significantly modified using the padj<0.05 threshold. **(b)** Volcano plot with a less stringent threshold of p-value<0.05, derived from the same dataset.
(c) Distribution of the modified ATAC-seq peaks among genomic region subtypes.

a

Pathway ID	Description	p.adjust	Gene ID
GO:2001233	regulation of apoptotic signaling pathway	0.0000428	IL1B/CD74/ATAD5/LGALS3/HMGB2/CTSH/CAV1/ICAM1/TNFSF12/LRRK2/MMP9/SKIL/BRCA1/RTKN2/IL1A/TNFAIP3/UNC5B/HMOX1/TNF/MYC/CD44/NUPR1/PTGS2/DDIAS/WFS1/VNN1/FGF10/TNFSF10/TRAF1/TREM2/NRP1/PLAUR/ADORA2A/SERPINE1/THBS1/S100A8/MIR21
GO:0097193	intrinsic apoptotic signaling pathway	0.0000904	CD74/AEN/ATAD5/EDA2R/CAV1/HIC1/LRRK2/CYP1B1/MMP9/SKIL/BRCA1/RTKN2/E2F1/HMOX1/TNF/MYC/BCL2A1/CD44/NUPR1/MELK/PTGS2/DDIAS/WFS1/VNN1/TP73/TREM2/PLAUR/ERN2/PPP1R13B/S100A8/MIR21
GO:2000106	regulation of leukocyte apoptotic process	0.0001441	CD74/LGALS3/CDKN2A/SLC7A11/JAK3/MERTK/NOD2/ADAM8/BCL6/AURKB/LILRB1/PDCD1/IL7R/CCR7/SLC46A2
GO:0043281	regulation of cysteine-type endopeptidase activity involved in apoptotic process	0.0008651	ANP32B/CTSH/HMGB1/NLRP1/TFAP4/MMP9/CLEC7A/SERPINB9/TNFSF15/TNF/MYC/CD44/BIRC3/PTGS2/NLRC4/TNFSF10/PLAUR/ADORA2A/KLF4/THBS1/NLRP2/S100A8
GO:1902041	regulation of extrinsic apoptotic signaling pathway via death domain	0.0022402	LGALS3/HMGB2/ICAM1/SKIL/BRCA1/TNFAIP3/HMOX1/SERPINE1/THBS1
GO:0043277	apoptotic cell clearance	0.0025867	HMGB1/TYROBP/C3/LRP1/TYRO3/MERTK/XKR5/TREM2/THBS1
GO:0072332	intrinsic apoptotic signaling pathway by p53 class mediator	0.0053792	CD74/AEN/ATAD5/EDA2R/E2F1/MYC/CD44/NUPR1/TP73/PPP1R13B/MIR21
GO:0070269	pyroptosis	0.0011236	GZMA/NLRP1/ELANE/GZMB/AIM2/NLRC4/TREM2
GO:1901214	regulation of neuron death	0.0004080	FCGR2B/IL6ST/ITGAM/GRN/GPNMB/TYROBP/PRNP/LRRK2/TYRO3/KIF14/C1QA/SLC7A11/UNC5B/HMOX1/TIGAR/ADAM8/TNF/APOE/JUN/NUPR1/XRCC2/CSF1/TLR6/WFS1/CCL3/TREM2/NRP1/ADORA2A/NTRK1/FOS/NR4A3
GO:0036473	cell death in response to oxidative stress	0.0086364	LRRK2/CYP1B1/SLC7A11/TNF/MET/MELK/TLR6/PYCR1/VNN1/TREM2/NR4A3/MIR21

b

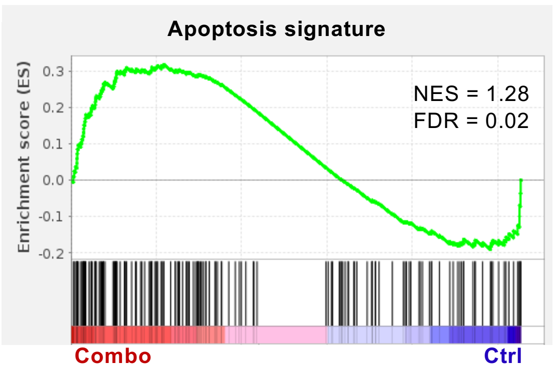

c

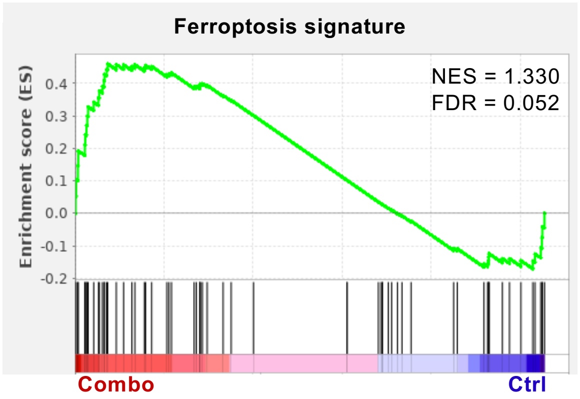

d

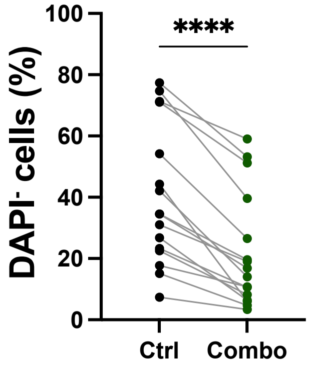

e

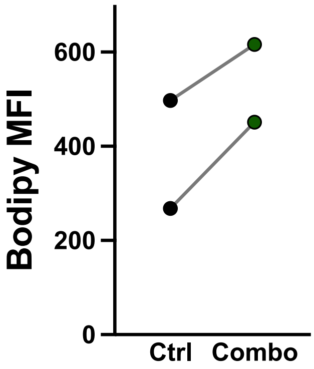

Appendix Figure S10. Upregulated cell death signatures and increased cell death in primary patient samples.

(a) List of significantly increased GO pathways related to cell death, by the combined palbociclib and TCP treatment.

(b-c) GSEA analysis of apoptosis and ferroptosis signatures of cells treated with the combination.

(d) Effects of the combination treatment on primary patient AML cell death. Primary AML patient cells (n=16) were treated with the Combination (Combo) or control media (Ctrl) for 96 hours. After treatment, live cells (DAPI negative) were quantified by flow cytometry. ****p= 7.71×10^{-7}.

(e) Lipid peroxidation was analyzed by flow cytometry on two patient samples using Bodipy C11 staining. p=0.0665.
