# Supplementary material for: Dual targeting of CDK6 and LSD1 is synergistic and overcomes differentiation blockade in AML
Source: EMBO Mol Med. 2025 Aug 29;17(10):2632–60. doi: 10.1038/s44321-025-00296-2 (PMC12514269; doi:10.1038/s44321-025-00296-2)
Supplement: Supplementary file 7 — Expanded View Figures [file 44321_2025_296_MOESM7_ESM.pdf]

Expanded View Figures

A

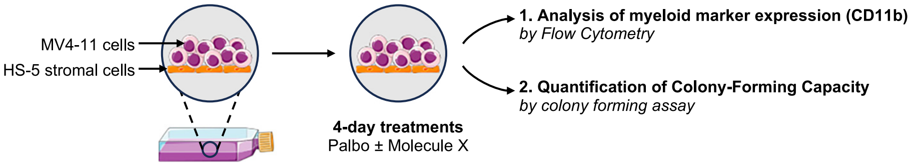

B

	CD11b (%)	
	Molecule alone	Molecule in combination with palbociclib
Vehicle control	*4,2*	-
Palbociclib	*21,6*	-
8-CPT	4,2	13,8
AC220	3,4	10,7
AGI5798	0,7	5,5
AGI6780	0,6	5,3
Arsenic trioxide	2,3	20,7
ATRA	11,1	51,3
Azacitidine	3,1	5
Calcitriol	32,5	74,3
Decitabine	10,4	7
Entinostat	1,8	10,6
OTX015	2,8	12,1
Panobinostat	1,6	9,8
Prima-1Met	0,2	10
Ruxolitinib	0,2	2
SGI110	7,4	5,8
TCP	9,6	44,7
Valproic acid	35,8	41,3
Venetoclax	3,8	12,3
Vorinostat	0,9	6,7

C

Treatment	No. of CFU-L	% CFU-L
Control	277	100.0
Palbo	78	28.2
TCP	282	101.8
Palbo+TCP	27	9.7
ATRA	218	78.7
Palbo+ATRA	270	97.5

Figure EV1. Screening of 19 molecules in combination with palbociclib.

(**A**) Experimental design for the drug screening: MV4-11 cells cocultured with HS-5 stromal cells were pretreated for 24 h with either vehicle control or palbociclib, followed by the exposure to either the second molecule alone, or the combination of palbociclib and the second molecule for an additional 72 h. Cells were then harvested to assess the expression of the myeloid differentiation marker CD11b by flow cytometry and to quantify the minor compartment of colony-forming cells. (**B**) List of molecules used in the screen (first column), and results of the analyses of CD11b expression following monotherapies (second column) or combined treatments with palbociclib (third column). Heatmap was applied to highlight the increase in CD11b expression. (**C**) Results of the colony-forming cell assay for monotherapies involving TCP and ATRA or in combination with palbociclib. Colonies (CFU-L) are expressed as numbers (second column) and as the percentage of colonies relative the control cells (last column). Heatmap was used in the table to highlight the drop in colony number.

© The Author(s)

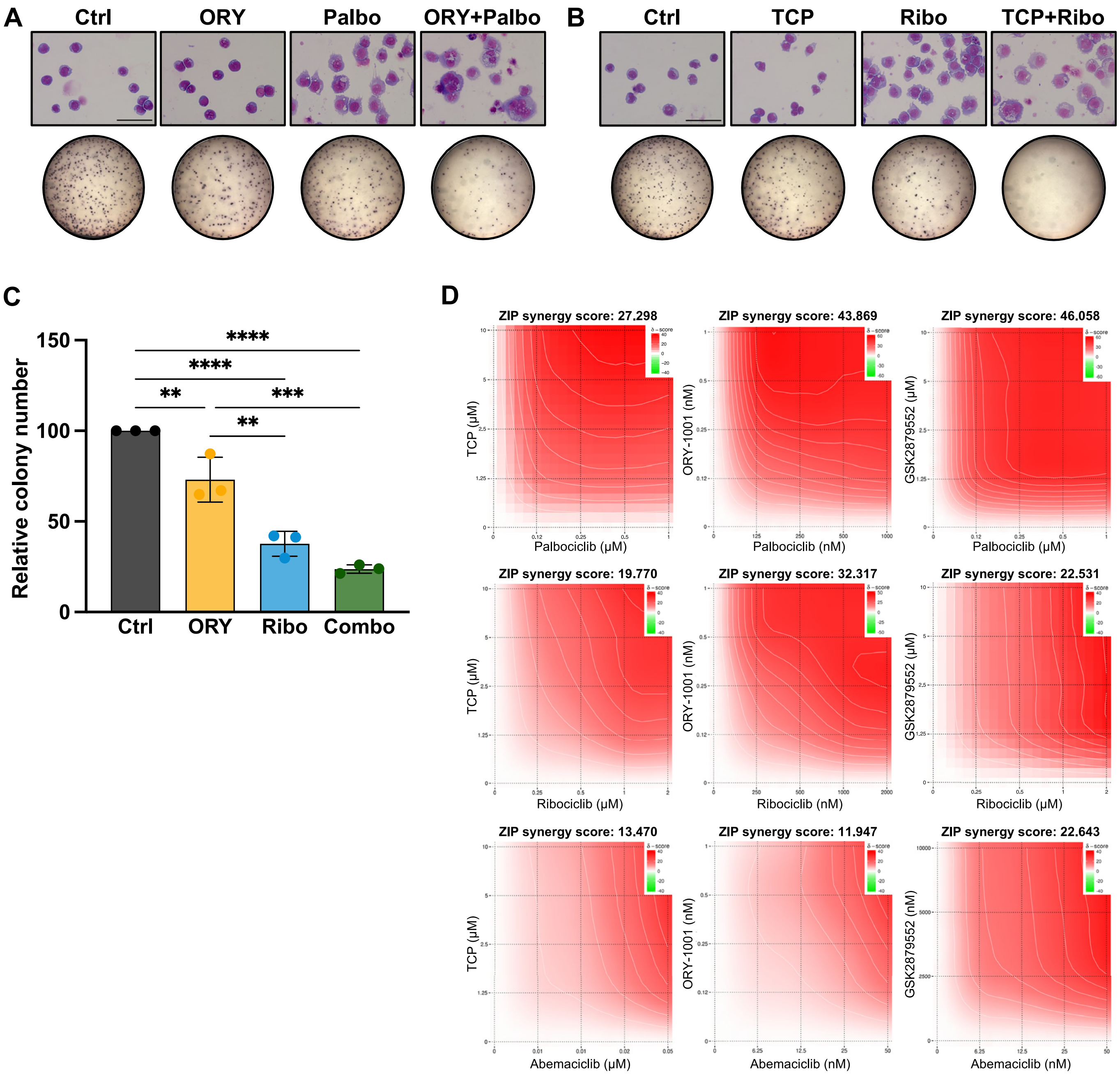

Figure EV2. Dual targeting of CDK6 and LSD1 is synergistic.

(**A**, **B**) Alternative LSD1 or CDK6 inhibitors. MV4-11 cells were treated with the LSD1 inhibitor ORY-1001 (ORY) with palbociclib (**A**) or with TCP and the CDK6 inhibitor ribociclib (Ribo) (**B**). After the 4-day treatment, cells were stained with May-Grünwald–Giemsa (top panel), and their capacity to form colonies was evaluated (bottom panel). (**C**) MV4-11 cells cocultured with HS-5 stromal cells were incubated with CDK6 inhibitor ribociclib at 1 µM, with ORY-1001 at 0.5 nM, or with the combination of ribociclib and ORY-1001 (Combo) for 96 h. After the treatment, leukemic cells were seeded in equal numbers in methylcellulose for 10 days. Data represent the mean ± SD of $n = 3$ independent experiments. Statistical analyses were performed using a one-way ANOVA followed by Tukey's test. $^{**}p < 0.01$, $^{***}p < 0.001$, $^{****}p < 0.0001$. Adjusted pvalues: Ctrl vs ORY $p = 0.0075$, Ctrl vs Ribo $p < 0.0001$, Ctrl vs Combo $p < 0.0001$, ORY vs Ribo $p = 0.0018$, ORY vs Combo $p = 0.0003$. (**D**) Evaluation of the synergy of CDK6 and LSD1 inhibitor combinations. MV4-11 cells were treated with increased concentrations of CDK6 inhibitor (palbociclib 0.125 to 1 µM; ribociclib 0.25 to 2 µM; abemaciclib 6.25 to 50 nM) in combination with LSD1 inhibitor (TCP 1.25 to 10 µM; ORY-1001 0.125 to 1 nM; GSK2879552 1.25 to 10 µM) for 96 h. The percentage of cells expressing CD11b was quantified by flow cytometry. Results were analyzed using the SynergyFinder tool, and represented by 2D contour plots. The red color indicates synergy.

 © The Author(s)

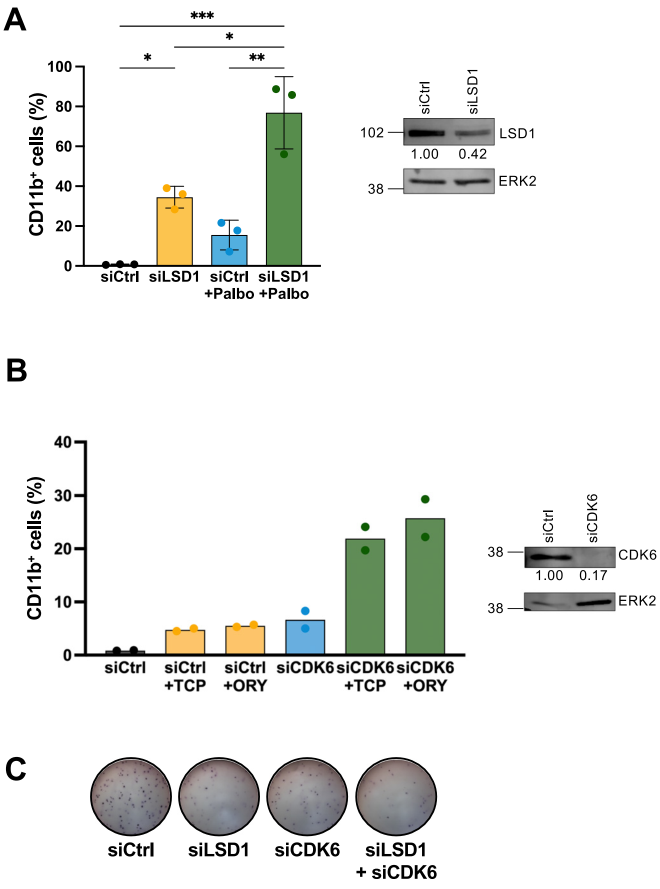

Figure EV3. Targeting of LSD1 and CDK6 by RNA interference recapitulated results obtained with small-molecule inhibitors.

(**A**) MV4-11 cells transfected with control (Ctrl) or LSD1 siRNAs were incubated with palbociclib (0.5 µM), as indicated, for 4 days. The percentage of CD11b-expressing cells was assessed by flow cytometry (left panel). Data represent the mean ± SD of $n = 3$ independent experiments. LSD1 protein expression was quantified by Western blot analysis (right panel); one of three experiments is shown. Statistical analyses were performed using a one-way ANOVA followed by Tukey's test. $^*p < 0.05$, $^{**}p < 0.01$, $^{***}p < 0.001$. Adjusted pvalues: siCtrl vs siLSD1 $p = 0.0363$, siCtrl vs siLSD1+Palbo $p = 0.0006$, siLSD1 vs siLSD1+Palbo $p = 0.0131$, siCtrl+Palbo vs siLSD1+Palbo $p = 0.0020$. (**B**) MV4-11 cells transfected with control (Ctrl) or CDK6 siRNAs were incubated with 5 µM TCP or 0.5 nM ORY-1001, as indicated, for 4 days. The percentage of CD11b-expressing cells was assessed by flow cytometry (left panel). Data represent the mean ± SD of $n = 2$ independent experiments. CDK6 protein expression was quantified by Western blot. One representative experiment out of two is shown (right panel). (**C**) MV4-11 cells transfected with control, CDK6 and/or LSD1 siRNAs were seeded on methylcellulose. CFCs were analyzed at day 10 post plating.

© The Author(s)

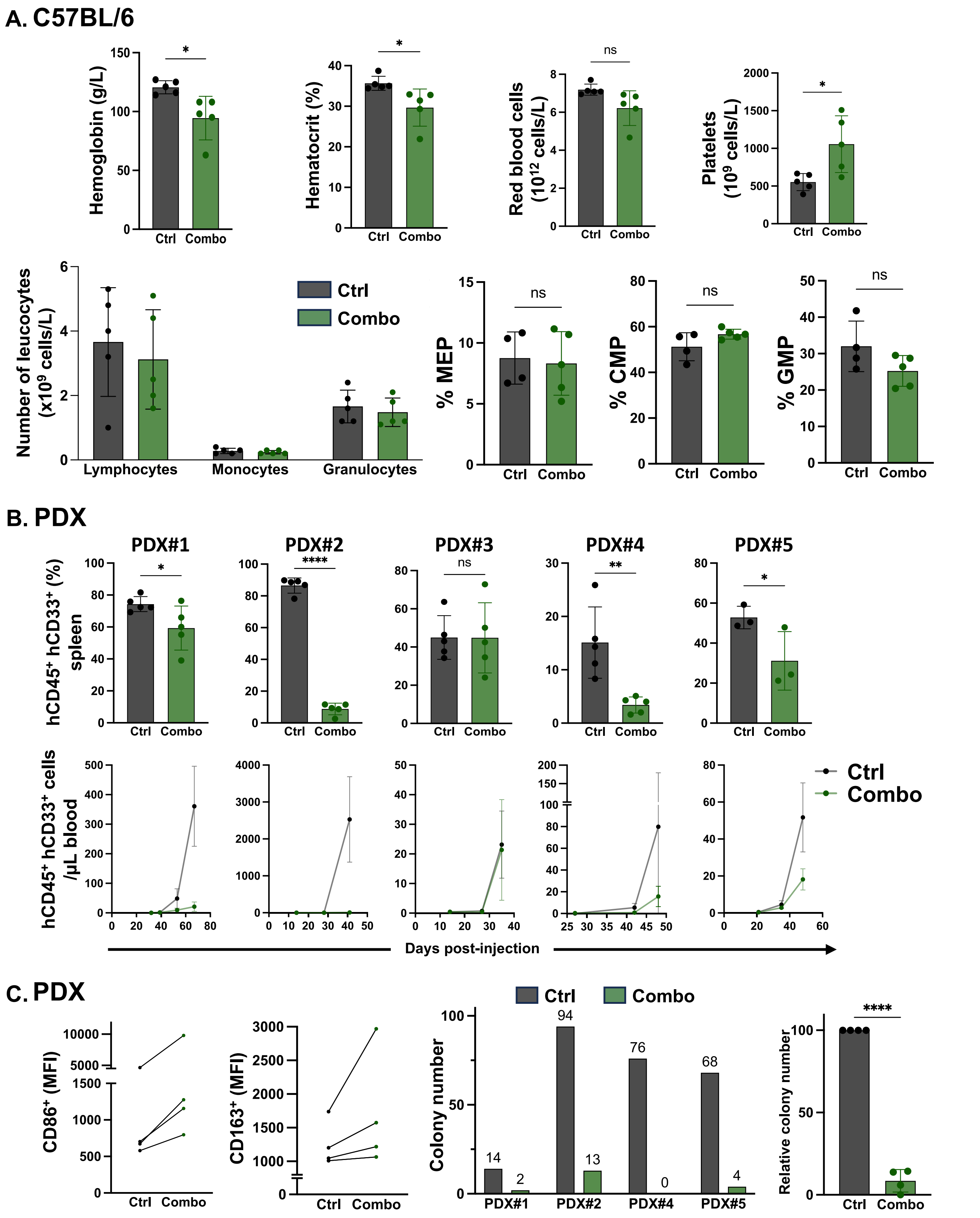
A. C57BL/6
Hemoglobin (g/L)
Hematocrit (%)
Red blood cells (10^{12} cells/L)
Platelets (10^9 cells/L)
Ctrl Combo
*
ns
Number of leucocytes ($x10^9$ cells/L)
Lymphocytes
Monocytes
Granulocytes
Ctrl
Combo
% MEP
% CMP
% GMP
B. PDX
PDX#1
PDX#2
PDX#3
PDX#4
PDX#5
hCD45+ hCD33+ (%) spleen
****
**
hCD45+ hCD33+ cells /µL blood
Days post-injection
C. PDX
CD86+ (MFI)
CD163+ (MFI)
Colony number
Relative colony number
14
2
94
13
76
0
68
4

Figure EV4. Analyses of in vivo effects of the combination on C57BL/6 and AML PDX mice.

(**A**) Evaluation of the combined treatment on mouse hematopoiesis. C57BL/6 mice were treated with intraperitoneal injections of the vehicle control or a combination of palbociclib (25 mg/kg) and ORY-1001 (0.0125 mg/kg), 4 days a week for 4 weeks. At the end of the treatments, blood counts and the bone marrow myeloid progenitors were analyzed. Data represent the mean ± SD of $n = 5$ mice. Statistical analysis was performed using an unpaired two-tailed Student's t-test. ns is not significant. *$p < 0.05$. CMP common myeloid progenitors, GMP granulocyte-monocyte progenitors, MEP megakaryocyte-erythroid progenitors. Top panels, $p = 0.0163$, $p = 0.0254$, $p = 0.0519$, and $p = 0.0211$. Bottom panels, $p = 0.6117$, $p = 0.4861$, $p = 0.8805$, $p = 0.7955$, $p = 0.0995$, and $p = 0.1129$. (**B**) Quantification of hCD45 + hCD33+ leukemic blast cells in the spleen and blood of the PDX NSG mouse models, as shown in Fig. 3D. PDX#1 $n = 5$, PDX#2 $n = 5$, PDX#3 $n = 5$, PDX#4 $n = 5$, PDX#5 $n = 3$. Statistical analysis was performed using an unpaired one-tailed Student's t-test. *$p < 0.05$, **$p < 0.01$, and ****$p < 0.0001$. (**C**) Analysis of the leukemic cells remaining following in vivo treatment of PDX mouse models. (Left) The expression of CD86 and CD163 late myeloid markers were analyzed on the remaining leukemic cells (detected as hCD45+ cells), for each untreated and matched PDX treated pair. (Middle and Right) Evaluation of the number of CFC progenitors remaining in the bone marrow of four independent PDX samples. Cells were seeded in methylcellulose in equal numbers for 10 days. The right histogram combined results from the four PDX, expressed as a percentage of colonies obtained relative to the control cells. Data represent the mean ± SD of $n = 4$ samples per condition. Statistical analysis was performed using a paired one-tailed Student's t-test, ****$p < 0.0001$.

© The Author(s)

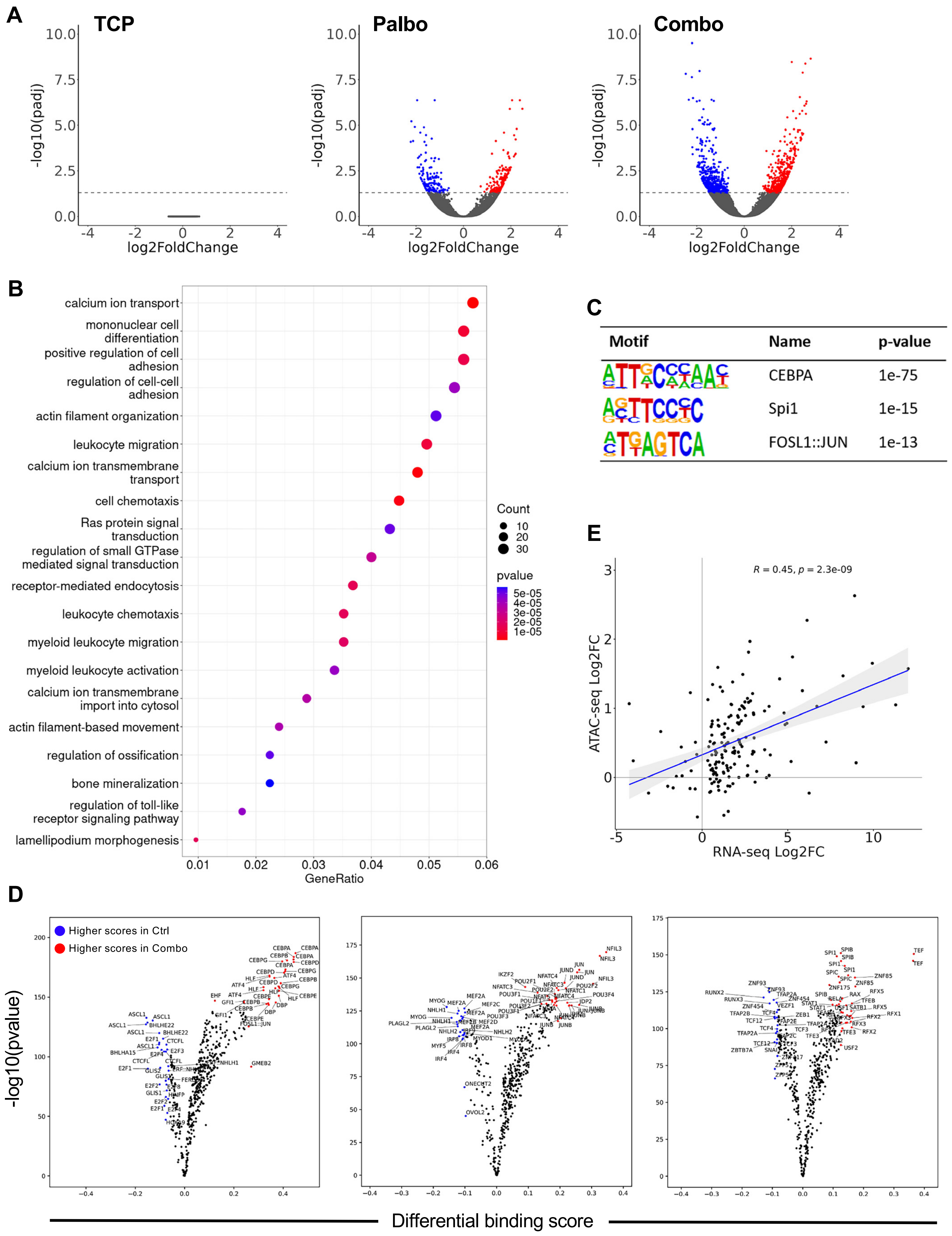
A
TCP
Palbo
Combo
-log10(padj)
log2FoldChange
B
calcium ion transport
mononuclear cell differentiation
positive regulation of cell adhesion
regulation of cell-cell adhesion
actin filament organization
leukocyte migration
calcium ion transmembrane transport
cell chemotaxis
Ras protein signal transduction
regulation of small GTPase mediated signal transduction
receptor-mediated endocytosis
leukocyte chemotaxis
myeloid leukocyte migration
myeloid leukocyte activation
calcium ion transmembrane import into cytosol
actin filament-based movement
regulation of ossification
bone mineralization
regulation of toll-like receptor signaling pathway
lamellipodium morphogenesis
GeneRatio
Count
pvalue
C
Motif
Name
p-value
CEBPA
1e-75
Spi1
1e-15
FOSL1::JUN
1e-13
E
R = 0.45, p = 2.3e-09
ATAC-seq Log2FC
RNA-seq Log2FC
D
Higher scores in Ctrl
Higher scores in Combo
-log10(pvalue)
Differential binding score

 © The Author(s)

Figure EV5. Analysis of chromatin accessibility of MV4-11 cells.

(**A**) Volcano plot representing modifications of chromatin accessibility assessed by ATAC-seq ($p_{adj} < 0.05$), of MV4-11 cells treated with the combination or the single molecules, compared with the control cells, as indicated ($n = 2$). (**B**) Gene Ontology biological processes modified by the combination treatment, corresponding to significantly deregulated chromatin regions. Statistical analysis was done using a hypergeometric test. (**C**) Analysis of enriched motifs in genomic regions showing significantly increased chromatin accessibility by the combination treatment. (**D**) Footprint analysis of ATAC-seq data for the combination treatment. Volcano plots indicate the transcription factor footprints that are most significantly altered following combination treatment. Transcription factors were classified alphabetically and divided among the three volcano plots. Statistical significance was assessed using Wald test, and the resulting p values were adjusted for multiple testing using the Benjamini–Hochberg procedure to control the false discovery rate. (**E**) Correlation plot of combined ATAC-seq and RNA-seq data. Significant genes in both RNA-seq and ATAC-seq analyses were plotted. Statistical analysis was performed using the Pearson correlation test.

© The Author(s)
